# Optic flow detection is not influenced by visual-vestibular congruency

**DOI:** 10.1371/journal.pone.0191693

**Published:** 2018-01-19

**Authors:** Vivian Holten, Paul R. MacNeilage

**Affiliations:** 1 German Center for Vertigo and Balance Disorders, University Hospital of Munich, Ludwig Maximilian University, Munich, Germany; 2 Department of Psychology, Cognitive and Brain Sciences Program, University of Nevada, Reno, Nevada, United States of America; University of Alberta, CANADA

## Abstract

Optic flow patterns generated by self-motion relative to the stationary environment result in congruent visual-vestibular self-motion signals. Incongruent signals can arise due to object motion, vestibular dysfunction, or artificial stimulation, which are less common. Hence, we are predominantly exposed to congruent rather than incongruent visual-vestibular stimulation. If the brain takes advantage of this probabilistic association, we expect observers to be more sensitive to visual optic flow that is congruent with ongoing vestibular stimulation. We tested this expectation by measuring the motion coherence threshold, which is the percentage of signal versus noise dots, necessary to detect an optic flow pattern. Observers seated on a hexapod motion platform in front of a screen experienced two sequential intervals. One interval contained optic flow with a given motion coherence and the other contained noise dots only. Observers had to indicate which interval contained the optic flow pattern. The motion coherence threshold was measured for detection of laminar and radial optic flow during leftward/rightward and fore/aft linear self-motion, respectively. We observed no dependence of coherence thresholds on vestibular congruency for either radial or laminar optic flow. Prior studies using similar methods reported both decreases and increases in coherence thresholds in response to congruent vestibular stimulation; our results do not confirm either of these prior reports. While methodological differences may explain the diversity of results, another possibility is that motion coherence thresholds are mediated by neural populations that are either not modulated by vestibular stimulation or that are modulated in a manner that does not depend on congruency.

## Introduction

As we move through the environment, the projected image of objects in the outside world drifts across the retina. This so-called optic flow provides information about the three-dimensional structure of the environment [1–3], the direction of self-motion [4, 5] and the distance travelled [6]. Besides the visual system, the vestibular system also contributes to self-motion perception in space. The semicircular canals and otolith organs of the inner ear sense linear accelerations and rotations of the head [7–9], thereby encoding motion of the head and its orientation relative to space [10].

Previous studies have shown that the activity of visual cortical areas processing self-motion information can be influenced by vestibular stimulation. For example, neurons of the dorsal medial superior temporal area (MSTd) in the visual cortex not only respond to visual optic flow patterns [11–14] but also to inertial motion [15–18]. The activity of some MSTd neurons correlates with an increased perceptual sensitivity of monkeys during a heading discrimination task when visual and vestibular cues were combined [19]. There are also indications that vestibular stimulation can influence visual perception in humans. For instance, locomotion decreases visual speed perception [20] and the motion aftereffect (MAE) decreases when observers adapt to expanding optic flow while moving forward [21]. From the above-mentioned studies it is clear that interactions between the visual and vestibular system exist. This raises the question of how these visual-vestibular interactions depend on whether or not the visual and vestibular stimulation directions match.

One would expect a sensitivity difference between matching and non-matching visual-vestibular stimuli based on the predictive coding framework, which suggests that the brain uses probabilistic information to interpret sensory signals and infer the state of the world [22–25]. In daily life, we are predominantly exposed to congruent rather than incongruent visual-vestibular stimulation. Because vestibular signals are associated with an increased likelihood of observing congruent optic flow, they may lead to increased sensitivity in detecting congruent compared to incongruent optic flow. Prior studies investigating the sensitivity to congruent versus incongruent optic flow during vestibular stimulation reported contradictory results. These studies presented expanding or contracting optic flow stimuli while the observer was moving forward or backward. Optic flow sensitivity was measured by manipulating the motion coherence of the random dot optic flow stimulus, which is the percentage of dots moving in a manner consistent with the global optic flow pattern versus the percentage moving randomly. Coherence percentage was manipulated from trial to trial to find the motion coherence that was just detectable, i.e. the threshold. One study [26] reported lower detection thresholds for optic flow patterns that were congruent with the vestibular stimulation, while another study [27] reported lower thresholds when the optic flow pattern was incongruent with the vestibular stimulation.

Here we have conducted a similar study investigating sensitivity during fore-aft movement in an attempt to resolve the conflict between prior reports. In addition, we have investigated whether there is an effect of visual-vestibular congruency on sensitivity for laminar (leftward or rightward) optic flow when observers simultaneously translate to the left or right. As described above, we expected lower detection thresholds for optic flow congruent with vestibular stimulation in both experiments, since observers experience congruent stimulation most often in daily life.

## Methods

### Observers

Fourteen observers (8 females, 6 males; age range 25–56, mean age: 31), including one author, participated in Experiment 1 (radial optic flow) and fifteen observers (9 females, 6 males; age range 25–55, mean age: 31.3), including the two authors, participated in Experiment 2 (laminar flow). Eleven of the observers participated in both experiments. All had normal or corrected-to-normal visual acuity. Except the authors, all observers were naïve to the purpose of the study. This was important, since otherwise observers could respond according to the hypothesis tested. Written informed consent, approved by the University Hospital Munich, was obtained from all observers and the purpose of the study was not concealed during the informed consent procedure. The experiment was conducted according to the principles expressed in the Declaration of Helsinki and approved by the ethics committee of the University Hospital Munich.

### Stimuli & apparatus

Observers were seated in a padded race seat mounted on a 6 degree-of-freedom motion platform (MOOG 6DOF2000E). The back of the observer’s head was positioned against a vacuum pillow and head movements were restrained by two head pads that were placed on the top front of the head. To mask the sound produced by the platform, observers wore noise-cancelling headphones (Bose QuietComfort 15) playing white noise during each trial of the experiment.

To be able to compare the results of the current study with the results of prior research, we used a stimulus design that was as close as possible to those prior studies. Therefore, we examined optic flow detection instead of optic flow discrimination, used an optic flow stimulus with a short presentation duration and without a speed gradient, and presented the optic flow stimuli in cardinal (forward/backward, left/right) directions.

Visual stimuli were presented on a 46” JVC (GD-463D10) full HD 3D LCD monitor (refresh rate 60 Hz, resolution 1920 x 1080 pixels) mounted on the motion platform. Stimuli subtended 93.4° by 67° and were viewed from a distance of ~43 cm. They were composed of 1500 randomly placed white dots (diameter 0.15°) on a black background, resulting in a dot density of 0.24 dots/deg^2^. The dots had an unlimited lifetime. In Experiment 1, signal dots of the radial optic flow stimulus (radius of annulus 46.7°) were either moving outward (expanding) or inward (contracting) with a constant angular speed of 3°/s. Expansion/contraction stimuli without speed gradients are not naturalistic ([28]), but they have been used in the literature before (e.g. [29–31]) and simplify comparisons between radial and laminar flow conditions. Such a single speed optic flow pattern results in stable and low motion coherence thresholds [32]. Noise dots were moving with the same angular speed in a randomly determined direction. Dots reaching the border of the annulus were replaced at a random location within the annulus. The central part (radius 5.9°) of the stimulus only contained the black background with the fixation dot (diameter 0.6°). The second experiment was similar to Experiment 1. However, signal dots either moved leftward or rightward with a constant angular speed of 3°/s. Signal dots reaching the edge of the screen were randomly replaced at the other side of the screen, while noise dots appeared at a random location. A fixation dot (diameter 0.6°) was presented at the center of the screen to prevent pursuit eye movements that occur when observers track the translating dots of the stimulus. To prevent the fixation dot from generating induced motion, a horizontal black bar (93.4° by 5.2°) was presented behind the fixation dot. This horizontal bar occluded some dots of the translating stimulus.

### Procedure

Three experimental conditions were used in the experiments. In the static condition, which served as a control condition, visual motion (Exp 1: expanding or contracting radial flow, Exp 2: leftward or rightward laminar flow) was presented on the screen while the motion platform remained stationary. In the congruent condition, the visual motion direction was consistent with the direction of vestibular stimulation (e.g. expanding visual motion with forward platform motion). In the incongruent condition, the direction of visual motion was inconsistent with the vestibular stimulation (e.g. expanding visual motion with backward platform motion).

**Fig 1:**
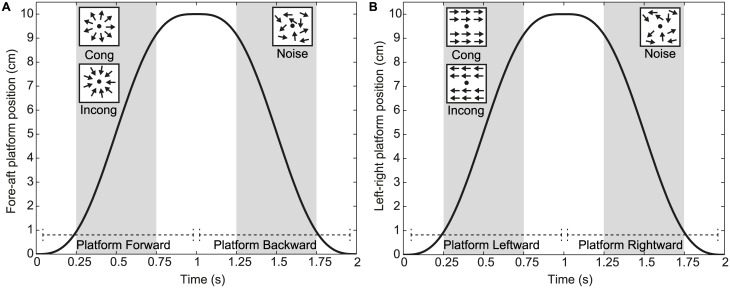
Schematic overview of trials used in the experiments. Both panels (A, B) represent the time course (x-axis) of the platform position (y-axis) used in the experiments. In both experiments, each experimental trial comprises two intervals lasting from 0-1s and 1-2s after platform motion onset respectively. In each interval, visual motion is presented (gray areas) between 0.25–0.75s after the start of the interval. It is therefore presented around the peak velocity of the vestibular stimulation. A. In experiment 1, radial optic flow that is either congruent (Cong) or incongruent (Incong) with the platform motion direction (forward) is presented in one interval, while in the other noise dots are presented. B. In experiment 2, leftward or rightward optic flow that is either congruent or incongruent with the platform motion (leftward) is presented in one interval, while in the other interval noise dots are presented. In both experiments, the presentation order of optic flow and noise dots intervals was counterbalanced across trials.

For each experimental condition, the motion coherence threshold was determined using a 3-down 1-up staircase procedure. Observers performed 100 trials per condition and since the staircases of all conditions were interleaved, the experiment consisted of 300 trials in total. An experimental trial consisted of two intervals. One of the intervals contained only noise dots and the other contained a combination of signal and noise dots. The observer’s task was to indicate which interval contained the signal dots. If the first interval contained signal dots, observers pressed the upper button of a button box that they held in their hands. When the second interval contained signal dots, observers pressed the lower button. The interval that was presented first (noise or signal) and the experimental condition (static, congruent, incongruent) were presented in a counterbalanced pseudorandom order. Motion direction was reversed between first and second intervals so that each trial started and ended in the same position (Fig 1). In this way, observers could not predict the linear self-motion direction of consecutive trials.

The motion coherence level was under staircase control, started at 90% (90% signal dots, 10% noise dots) and increased or decreased with steps of 5%. Observers started the experiment by pressing a button on the button box. Subsequently, the first interval started and the motion platform moved forward or backward for 1s (10cm) or remained stationary (Fig 1). The motion profile of the platform was a raised cosine profile, with a peak velocity of 20 cm/s. A central fixation point was displayed on the screen throughout each interval but visual motion was presented only between 0.25s and 0.75s after the motion platform onset. It was therefore presented around the peak velocity of the vestibular stimulation, which was at 0.5s after platform onset. In this way, the visual motion was not presented during slow (< 10 cm/s) platform movements, ensuring that the vestibular system was already activated before the visual motion started. After the first interval, the platform moved in the opposite direction for 1s. During this time period, the second interval was presented with a similar motion sequence as the first interval. So, first a 0.25s presentation of the fixation dot, followed by the presentation of visual motion and a fixation dot for 0.5s and then again a 0.25s presentation of the fixation dot alone. After the second interval finished, the fixation dot turned green to indicate that observers could give a response. After each trial, observers started a new trial by pressing a button. This allowed observers to take a short break between trials if needed.

### Analysis

For each observer and each condition, the proportion of correct responses was calculated per coherence level and a cumulative Gaussian function was fit to the data using the PAL_PFML_Fit function of the Palamedes Toolbox for Matlab. The lapse rate parameter (lambda) was set at 0.01. The coherence level where each Gaussian function crossed the 79.4% correct level was taken as the threshold. This value was chosen because it represents the value to which 3-down 1-up staircases converge [33], allowing for straightforward comparison of our threshold measures with prior studies [26, 27].

To examine coherence threshold differences between conditions, a one-factor repeated measures analysis of variance (ANOVA) was performed with the within-subject factor condition (3 levels: static, incongruent, congruent). To investigate whether the coherence threshold differed between motion directions, the data were separated based on motion direction. For each observer, this resulted in 50 trials per motion direction per condition and a cumulative Gaussian function was fit to these data to determine the coherence threshold. In case the psychometric function crossed the 79.4% correct level at a negative motion coherence level, the lowest motion coherence level tested was used as the threshold. This only happened for 7 of the 174 fits when the data were separated based on motion direction (Exp1: 14[observers]*2[motion directions]*3[conditions] fits, Exp2: 15*2*3 fits), and occurred when the slope of the Gaussian function was very shallow. For each motion direction, the coherence thresholds were collapsed over conditions (incongruent, congruent, stationary), resulting in a single threshold per motion direction per observer. A paired-samples t-test was performed to examine a significant difference between the motion directions. To determine whether any differences between motion directions were caused by vestibular stimulation, a 2 x 3 repeated measures ANOVA with the within-subject factors motion direction (expanding, contracting) and condition (static, incongruent, congruent) was performed. Partial eta squared was used to report effect sizes for the main and interaction effects. Pairwise comparisons with a Bonferroni correction were used to examine significant differences between conditions.

## Results

### Results of Experiment 1: Radial optic flow

#### Visual-vestibular congruency

**Fig 2:**
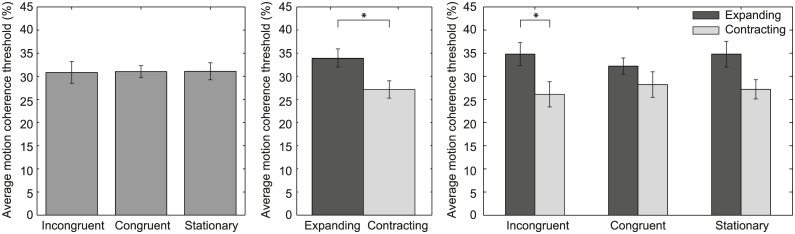
Average motion coherence threshold across observers for radial optic flow. In the left panel, the average motion coherence threshold across observers (y-axis) is depicted for the incongruent, congruent and stationary condition (x-axis). The center panel shows the average motion coherence threshold across observers for each visual motion direction (expanding, contracting optic flow). The right panel depicts the average motion coherence threshold across observers for the incongruent, congruent and stationary condition separated per motion direction. In all panels, error bars indicate ± 1 SEM. An asterisk indicates a significant difference between conditions.

The motion coherence threshold across observers is depicted per condition in the left panel of Fig 2. Surprisingly, our results show no difference in motion coherence threshold between conditions (F(2,26) = .011, *p* = .99, *η*_*p*_^2^ = .001), indicating that visual-vestibular congruency as well as vestibular stimulation in general does not affect the sensitivity to detect a radial optic flow pattern. To investigate whether these results were caused by groups of observers showing opposite results and thereby cancelling any overall effect, we calculated for each observer the bootstrapped confidence interval belonging to each condition. For each observer, the confidence intervals of the incongruent and congruent visual-vestibular stimulation conditions were overlapping, which shows that the lack of a congruency effect is not the result of groups of observers showing opposite results.

#### Motion direction

Our results show an effect of motion direction on the motion coherence threshold (center panel of Fig 2). The motion coherence threshold is lower for contracting than for expanding optic flow (t(13) = 2.57, *p* = .023). To examine whether this effect is caused by vestibular stimulation, the data were separated per condition and motion direction (right panel of Fig 2). A repeated-measures ANOVA revealed a main effect of motion direction (F(1,13) = 9.20, *p* = .010, *η*_*p*_^2^ = .41) and no main effect of condition (F(2,26) = .08, *p* = .920, *η*_*p*_^2^ = .01). Note that in Fig 2, a significant difference between expanding and contracting optic flow is only present for the incongruent condition. However, no interaction between motion direction and condition (F(2,26) = 1.02, *p* = 0.376, *η*_*p*_^2^ = .07) was observed. The lack of the interaction indicates that the lower motion coherence threshold observed for contracting optic flow is a general effect and not the result of vestibular stimulation.

### Results of Experiment 2: Laminar flow

#### Visual-vestibular congruency

**Fig 3:**
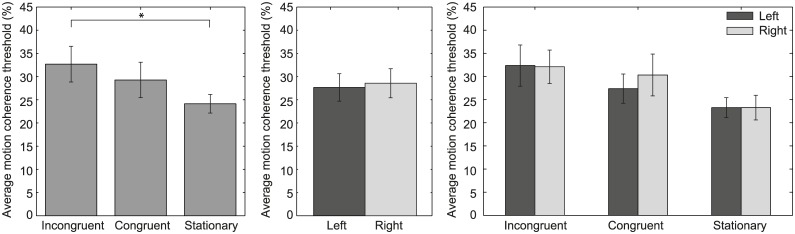
Average motion coherence threshold across observers for leftward/rightward optic flow. In the left panel, the average motion coherence threshold across observers (y-axis) is depicted for the incongruent, congruent and stationary condition (x-axis). The center panel shows the average motion coherence threshold across observers for each visual motion direction (leftward, rightward optic flow). The right panel depicts the average motion coherence threshold across observers for the incongruent, congruent and stationary condition separated per motion direction. In all panels, error bars indicate ± 1 SEM. An asterisk indicates a significant difference between conditions.

For each condition, the averaged motion coherence threshold across observers is depicted in the left panel of Fig 3. Results show a main effect of the stimulus condition on the motion coherence threshold (F(2,28) = 4.89, *p* = .015, *η*_*p*_^2^ = .26). To determine whether the main effect is caused by a difference in stimulus congruency or vestibular stimulation in general, additional statistical tests were performed. Further examination shows no difference in motion coherence threshold between incongruent and congruent visual-vestibular stimulation (t(14) = 1.32, *p* = .626, *r* = .33). As is the case for radial optic flow, this result is not caused by groups of observers showing opposite results, since on an individual observer level, all but one observer showed bootstrapped 95% confidence intervals that were overlapping between these conditions. Subsequently, we tested whether vestibular stimulation in general generated higher motion coherence thresholds than visual stimulation only. It turned out that vestibular stimulation generates a higher motion coherence threshold than the stationary (visual only) condition when the visual stimulation is incongruent with the vestibular stimulation (t(14) = 2.94, *p* = .032, *r* = .62) but not when it is congruent (t(14) = 1.86, *p* = .251, *r* = .45).

#### Motion direction

The center panel of Fig 3 shows for each motion direction the averaged motion coherence threshold across observers. In contrary to radial optic flow, no significant difference in motion coherence threshold is observed between leftward and rightward motion (t(14) = -.411, *p* = .688, *r* = .11). This difference is also absent when the motion coherence threshold belonging to each motion direction is analyzed separately per condition (right panel of Fig 3; F(1,14) = .169, *p* = .688, *η*_*p*_^2^ = .012). Also no interaction between the motion direction and the stimulus condition was observed (F(2,28) = 0.449, *p* = .642, *η*_*p*_^2^ = .031). This result shows that the motion coherence threshold was similar between leftward and rightward visual motion for all stimulus conditions (right panel of Fig 3). Moreover, it also shows that vestibular stimulation in general did not alter the motion coherence threshold for detecting leftward or rightward motion.

## Discussion

In the current study, we investigated whether optic flow sensitivity was influenced by the congruency of the vestibular stimulation. We expected observers to be more sensitive when optic flow was congruent rather than incongruent with vestibular stimulation, since congruent stimuli are experienced more often. If vestibular stimulation is associated with an increased likelihood of experiencing congruent optic flow and the nervous system makes use of this probabilistic association, detection performance should be better for congruent optic flow. However, our results do not support this hypothesis since detection thresholds did not differ between congruent and incongruent visual-vestibular stimulation for either radial or laminar optic flow. We conclude that vestibular stimulation does not impact coherence thresholds under the conditions of the current experiments and consider possible reasons that our results may differ from previous findings.

### Visual-vestibular congruency

Despite prior contradictory reports that visual-vestibular congruency can impact motion coherence thresholds [26, 27], we did not observe such an effect. Differences in results may be a consequence of differences in stimulus parameters. Table 1 provides an overview. The dot density, the stimulus duration and the maximum velocity of the vestibular stimulation are parameters that in our study have a value that is in between the values of the other studies. Since we do not find a congruency effect in our study, and the other studies report opposite effects, it might be that one of these stimulus parameters causes the effect to reverse direction. However, this seems unlikely.

**Table 1:** Overview of stimulus parameters used in the current study and two prior studies reporting contradictory findings.

	*Current Study*	*Edwards et al*. *(2010)*	*Shirai & Ichihara (2012)*
**Visual stimulus**			
Viewing	Binocular	Binocular	Monocular
Aperture	Annular outer 93.4°, inner 11.8°	Annular outer 82°, inner 5.9°	Circular subtending 20°
Dot density	0.24 dots/deg^2^	0.03 dots/deg^2^	0.63 dots/deg^2^
Number of dots	1500	150	200
Dot speed	3°/s	19.4°/s	Dependent on wheelchair velocity (max 3.2°/s)
Dot size	0.15°	0.97°	0.3°
Speed gradient	Flat	Flat	Flat
Noise dots	Random direction motion	Random walk	Random direction motion
Dot lifetime	Unlimited	Unlimited	1 to 10 frames
Fixation dot	In center of aperture	In center of aperture	Not mentioned in text
Stimulus duration	500 ms	200 ms	2000 ms in total, 1000ms per motion direction
**Vestibular stimulation**			
Device	Moog 6DOF platform	Earthquake platform	Wheelchair
Motion	Raised cosine profile	Constant Acc of 8 cm/s	Sine wave movement
Max velocity	20 cm/s	11 cm/s	40 cm/s
Stimulation onset	250 ms platform movement before and after visual stimulation	300 ms platform movement before and after visual stimulation	Wheelchair movement onset simultaneous with visual motion onset
**Procedure**			
Intervals	2 (1 signal, 1 pure noise) Observer movement direction opposite in both intervals	2 (1 signal, 1 pure noise) Observer movement direction similar in both intervals	2 (1 signal, 1 pure noise) Forward and backward movement in each interval
Staircase	3-down, 1-up	3-down, 1-up	3-down, 1-up
Threshold	79.4% of psychometric function	Mean of 10 staircases (last 8 reversals)	Mean of 6 staircases (last 6 reversals)

Besides a difference in stimulus parameters, one might argue that eye movements caused by the vestibulo-ocular reflex or generated by tracking individual dots of the visual motion pattern could lead to the opposing findings between the studies, since eye movements affect the perceived speed of a visual motion pattern [34]. If perceived speed influences the motion coherence threshold, eye movements may have caused the conflicting findings since the current and prior studies did not measure eye movements. However, the motion coherence threshold does not depend on the velocity of a motion pattern [35, 36], so it is unlikely that eye movements are responsible for the varying results, particularly if observers maintained fixation on the fixation point as instructed. One might also question whether our experiments had enough statistical power [37] to show a visual-vestibular congruency effect. In our experiments, 14 and 15 observers participated respectively, and prior research used only 4 [26] or 11 observers [27]. A lack of statistical power therefore cannot explain our current results.

While the two studies described above report an effect of visual-vestibular congruency, other studies have failed to find such effects. For example, vestibular stimulation did not lead to biases in perception of congruent or incongruent optic flow in a binocular rivalry paradigm [38]. Similarly, Shirai and Ichihara (2012) failed to observe an effect of congruency on coherence thresholds for vertically moving optic flow patterns presented during fore-aft movement with the head facing downwards, i.e. toward the ground.

We hypothesized that congruent stimuli would be more easily detected, but it is also reasonable to hypothesize that deviations from congruency (i.e. incongruent, less common stimuli) might be easier to detect. This would be analogous to postural sway observations, where observers show more sway to less common optic flow patterns, such as non-rigid optic flow patterns [39]. However, we did not find evidence to support this hypothesis either.

The lack of congruency effects in general, may be due to the heterogeneity of optic flow patterns in everyday life. Our definition of congruency does not explicitly account for possible effects of eye movements. During natural behavior, eye movements greatly impact optic flow at the retina, thereby complicating the mapping between retinal and vestibular signals ([40]). Further heterogeneity will result from differences in environmental structure. For example, moving towards a wall results in an optic flow pattern with a linear speed gradient, whereas moving inside a tunnel results in a quadratic speed gradient. Another possibility is that perceptual thresholds are not the best method to assess congruency effects. Behavioral measures such as postural or oculomotor responses may be dependent on visual-vestibular congruency even if perception is not.

Overall, our results cast doubt on the generality of previous findings, and suggest that the effect of vestibular congruency on optic flow thresholds is not robust. The lack of a congruency effect may be supported on a neuronal level. In macaques, it is known that the number of neurons that are sensitive to congruent or incongruent combinations of visual-vestibular stimulation is about equal in some areas that process optic flow (MSTd [16], ventral intraparietal area (VIP) [41]). If this is also the case in humans, and these populations impact coherence thresholds, it could be an explanation why we do not observe a perceptual sensitivity difference between congruent and incongruent visual-vestibular stimulation for either radial or laminar optic flow.

### Visual motion direction

Results suggest that observers have lower coherence thresholds for contracting than expanding optic flow. This effect is independent of vestibular stimulation since it is also observed when only visual motion was presented. This finding agrees with the results of prior research that also reported a lower detection threshold for contracting than expanding optic flow when only visual motion was presented [32, 42–44]. A possible explanation of this anisotropy, also suggested by Edwards and Ibbotson (2007), is that a higher sensitivity to contracting optic flow could be important to maintain balance. Namely, since our feet project forwards, we can lean further forward without losing balance than backward; hence the higher sensitivity for contracting optic flow.

In contrast to the anisotropy reported for radial optic flow, the optic flow direction did not influence coherence thresholds for laminar optic flow. This finding is supported by the results of another study [45], which also reported no detection threshold difference between leftward and rightward motion. Also from a neuronal perspective, one would not expect an anisotropy, since no distribution bias between leftward or rightward motion has been found in the middle temporal (MT) area [46], which is involved in the processing of linear motion.

### Visual-vestibular stimulation versus visual only

During vestibular stimulation, the motion coherence threshold for radial and laminar optic flow is similar to or higher than during visual stimulation only. Based on Bayesian cue integration principles [47, 48], one might expect the opposite, i.e. that observers are more sensitive when two cues (e.g. visual and vestibular) are available compared to when a single cue is available (e.g. visual or vestibular). This finding suggests that not only is the vestibular signal not used, it may actually impair detection performance, regardless of whether or not it is congruent with the optic flow stimulus. A possible explanation for this effect is vibration of the observer’s head relative to the visual display during platform movement, despite head-stabilization measures, resulting in additional undocumented noise in the visual motion stimulus.

## Conclusion

Under the conditions of the current experiment, visual-vestibular congruency does not affect motion coherence thresholds for radial or laminar optic flow. These results raise doubts about the generality of previous findings, conducted with fewer subjects, reporting that congruency impacts coherence thresholds. While methodological differences may explain the diversity of results, another possibility is that motion coherence thresholds are mediated by neural populations that are either not modulated by vestibular stimulation or that are modulated in a manner that does not depend on congruency.
